# Dietary Leucine Supplement Ameliorates Hepatic Steatosis and Diabetic Nephropathy in db/db Mice

**DOI:** 10.3390/ijms19071921

**Published:** 2018-06-30

**Authors:** Kuan-Hsing Chen, Yi-Ling Chen, Hsiang-Yu Tang, Cheng-Chieh Hung, Tzung-Hai Yen, Mei-Ling Cheng, Ming-Shi Shiao, Jan-Kan Chen

**Affiliations:** 1 Kidney Research Center, Chang Gung Memorial Hospital, Chang Gung University, School of Medicine, Taoyuan 333, Taiwan; guanhsing3795@gmail.com (K.-H.C.); cchung9651@yahoo.com (C.-C.H.); m19570@adm.cgmh.org.tw (T.-H.Y.); 2 Department of Biomedical Sciences, College of Medicine, Chang Gung University, Taoyuan 333, Taiwan; tangshyu@gmail.com (H.-Y.T.); chengm@mail.cgu.edu.tw (M.-L.C.); msshiao@gap.cgu.edu.tw (M.-S.S.); 3 Healthy Aging Research Center, Chang Gung University, Taoyuan 333, Taiwan; 4 Metabolomics Core Laboratory, Chang Gung University, Taoyuan 333, Taiwan; 5 Clinical Phenome Center, Chang Gung Memorial Hospital, Linkou, Taoyuan 333, Taiwan; 6 Department of Physiology, College of Medicine, Chang Gung University, Taoyuan 333, Taiwan; ndzionyi@msn.com

**Keywords:** leucine, hepatic steatosis, diabetic nephropathy, db/db mice, metabolomics

## Abstract

Dietary leucine supplementation has been explored for the therapeutic intervention of obesity and obesity-induced metabolic dysfunctions. In this study, we aim to examine the effects of dietary leucine supplementation in db/db mice. Mice were treated with or without leucine (1.5% *w*/*v*) in drinking water for 12 weeks. The leucine supplement was found to reduce insulin resistance and hepatic steatosis in db/db mice. Using Nuclear Magnetic Resonance (NMR)-based lipidomics, we found that the reduction of hepatic triglyceride synthesis was correlated with attenuated development of fatty liver. In addition, diabetic nephropathy (DN) was also ameliorated by leucine. Using liquid chromatography–time-of-flight mass spectrometry (LC-TOF MS)-based urine metabolomics analysis, we found that the disturbance of the tricarboxylic acid (TCA) cycle was reversed by leucine. The beneficial effects of leucine were probably due to AMP-activated protein kinase (AMPK) activation in the liver and kidneys of db/db mice. Thus, dietary leucine supplementation may potentially be a nutritional intervention to attenuate hepatic steatosis and early DN in type II diabetes.

## 1. Introduction 

Type 2 diabetes mellitus (T2DM) has emerged as one of the most severe health concerns in the world. The longitudinal progression of obesity, insulin resistance, metabolic syndrome, T2DM, and T2DM-associated complications, particularly cardiovascular disease and diabetic nephropathy (DN), contribute to a heavy burden on the social-economic health-care system. Obesity is often regarded as the initial step of this longitudinal progression, and it develops from an imbalance between energy intake and energy expenditure [1,2]. Diets, physical activity, and even social factors have been shown to impact genetic background and alter metabolic homeostasis [3,4]. Regarding diet, most attention has focused on how changes in macronutrient composition, i.e., the proportion of fat, carbohydrate, and protein, can affect metabolic risk. The branched chained amino acids (BCAA) leucine, isoleucine and valine have been shown to function as regulators of hormonal signaling in addition to serving as nutrients. In addition, baseline BCAA and aromatic amino acids (tyrosine and phenylalanine) have been shown to have significant associations with future diabetes development in a 12-year follow-up study [5]. Newgard et al. showed that metabolomics profiling obese versus lean humans demonstrated a BCAA-related metabolite signature, suggesting that increased catabolism of BCAA is correlated with insulin resistance [6]. Newgard et al. showed that in high-fat (HF) plus BCAA mixture-fed rats, food intake and body weight gain were both reduced compared with that of HF-fed rats. However, insulin resistance was not improved by BCAA supplement and was ascribed to mTORC1 signaling pathway activation by BCAA [6]. More recent studies also demonstrated that a BCAA-restriction diet may promote metabolic health and improve insulin sensitivity in both lean and obese mice [7,8,9].

By contrast, single leucine supplementation has been shown to block high-fat diet (HFD)-induced obesity, hyperglycemia, and dyslipidemia in animal models [10,11]. In this regard, the use of dietary leucine supplementation has been extensively explored as the therapeutic intervention of obesity and obesity-induced metabolic dysfunctions, such as non-alcoholic fatty liver disease (NAFLD) and insulin resistance, despite its possible mTORC1 activation [11,12,13]. The possible mechanisms underlying leucine benefits have been suggested to correlate with improving insulin signaling and decreasing inflammation in adipose tissue, which led to multiple aspects of the metabolic benefits, such as AMP-activated protein kinase (AMPK) activation [14]. However, the detailed molecular mechanism is still unclear. In the T2DM mouse model, Guo et al. [15] showed that chronic leucine supplementation leads to better glycemic control, mitochondrial oxidative function, and reduced inflammatory profile. However, the possible beneficial effects of leucine supplementation on T2DM-related end-organ damage, such as DN and NAFLD, have not yet been investigated. As T2DM and associated end organs damage are important global health issues, it is of interest to explore if leucine supplementation can help to ameliorate these complications. In the present study, we evaluate the effects and explore the underlying mechanisms of dietary leucine supplementation in the amelioration of NAFLD and DN in db/db mice.

## 2. Results

### 2.1. Plasma Concentration of Leucine and Body Weight Change 

**Figure 1:**
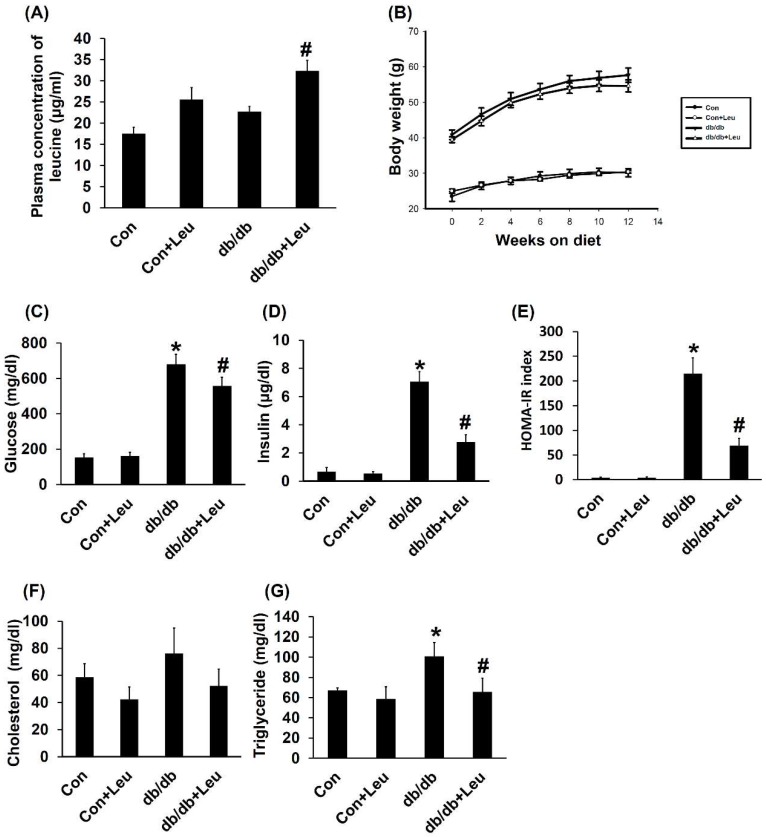
Plasma leucine concentration and insulin resistance measured by homeostatic model assessment-insulin resistance (HOMA-IR) were improved by leucine supplementation in db/db mice. Mice were sacrificed after 12 weeks of chew diet and normal drinking water. In mice treated with leucine, 1.5% *w*/*v* of leucine was added in the drinking water. (**A**) Plasma leucine concentration was measured by liquid chromatography-mass spectrometry (LC-MS) at week 12. (**B**) Body weights were monitored from week 0 to week 12 and were recorded biweekly. (**C**) Fasting glucose was measured in week 12 when mice were removed from metabolic cages. (**D**) Fasting mice insulin was measured in week 12 when mice were removed from metabolic cage. (**E**) HOMA-IR index was calculated by formula [HOMA-IR = (fasting glucose levels×fasting insulin levels)/22.5]. (**F**) Total plasma cholesterol levels were determined from mice 12 weeks after diet with leucine supplementation. (**G**) Plasma triglyceride levels were determined 12 weeks after diet with leucine supplementation. Data were presented as mean ± SEM, *n* = 5 in Con and Con + Leu groups; *n* = 6 in db/db and db/db + Leu groups. * *p* < 0.05, Con vs db/db mice; # *p* < 0.05, db/db vs db/db + Leu mice.

Daily leucine supplementation (1.5% *w*/*v*) in drinking water led to approximate 1.5-fold increase of plasma leucine concentrations (Figure 1A). The db/db mice and db/db + Leu mice were characterized by a much heavier body weight than the Con and Con + Leu groups (Figure 1B). However, db/db mice and db/db + Leu mice exhibited similar body weight increase regardless of leucine supplementation (Figure 1B).

### 2.2. Leucine Supplementation Reduced Insulin Resistance, and Plasma Triglycerides, with No Significant Effect on Plasma Cholesterol 

The db/db mice exhibited higher levels of blood glucose compared with other groups; levels were reduced by dietary leucine supplementation (Figure 1C). Leucine supplementation caused a more significant reduction in plasma insulin and homeostatic model assessment-insulin resistance (HOMA-IR) index in db/db + Leu mice compared with that of db/db mice (Figure 1D,E). In addition, plasma triglyceride levels were also decreased by approximately 35% in db/db + Leu mice after 12 weeks of leucine supplement (Figure 1G). However, leucine supplementation showed no significant effect on plasma cholesterol levels in db/db mice (Figure 1F).

### 2.3. Leucine Supplementation Attenuated Hepatic Lipid Accumulation 

**Figure 2:**
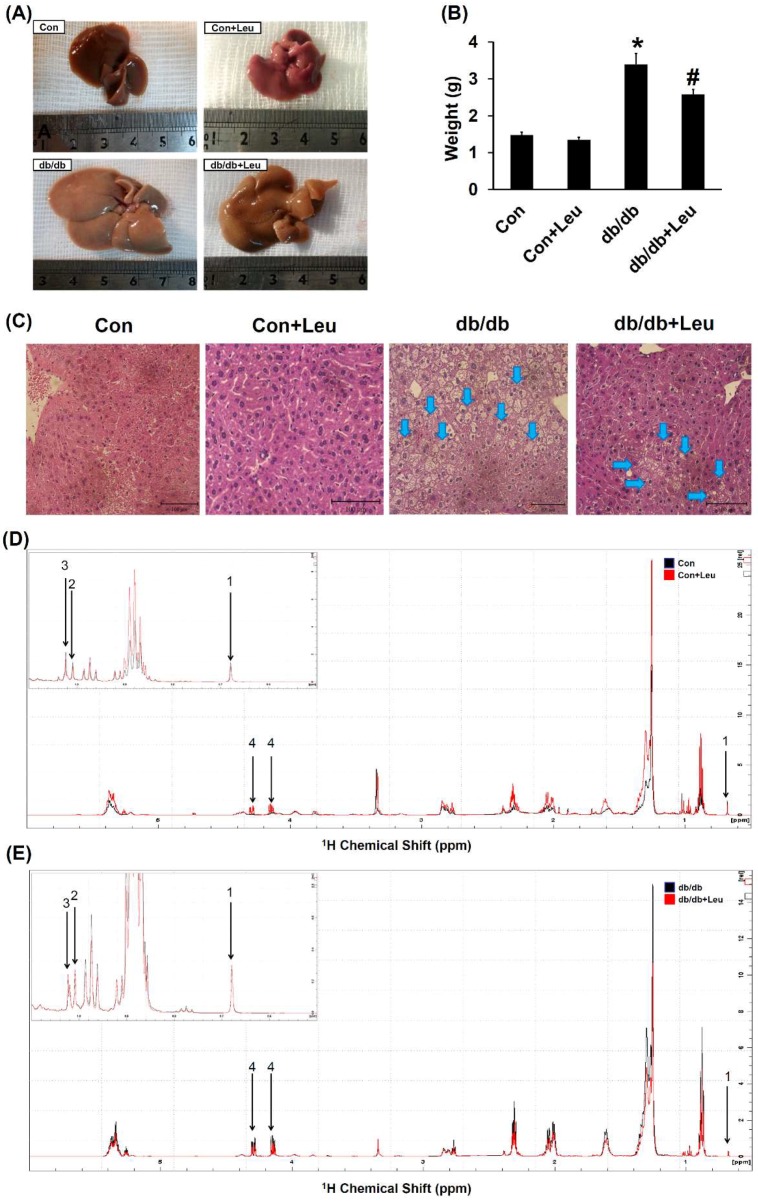
Leucine supplementation ameliorated hepatic steatosis in db/db mice. Mice were sacrificed, and livers were harvested after 12 weeks of chew diet and normal drinking water. In mice fed with leucine, 1.5% *w*/*v* of leucine was added in the drinking water. (**A**) The liver size and (**B**) weight of different groups of mice. (**C**) Livers were formalin-fixed and embedded in paraffin, and sections were stained with H&E stain. Arrows indicate macrovacuolar steatosis and the images were magnified 200×. Data were presented as mean ± SEM, *n* = 5 in Con and Con + Leu groups; *n* = 6 in db/db and db/db + Leu groups. * *p* < 0.05, Con vs. db/db mice; # *p* < 0.05, db/db vs. db/db + Leu mice. (**D**,**E**) Six hundred MHz ^1^H NMR spectra assignments with chemical shifts for signals identified in the liver lipid extract. The labeled peaks were: 1. total cholesterol (C-18, CH3); 2. free cholesterol (C-19, CH3); 3. esterified cholesterol (C-19, CH3); 4. Triglyceride (TG) (C-1/C-3, CH2).

The liver weight was significantly increased in db/db mice compared with that of control mice. Also, the liver weight was significantly reduced in db/db + Leu mice compared with that of db/db mice (Figure 2A,B). Liver tissue from db/db mice showed a uniform macrovacuolar steatosis, which refers to a single large cytoplasmic lipid vacuole displacing the nucleus to the periphery of the hepatocyte. The macrovacuolar steatosis was significantly attenuated in the liver of leucine-treated db/db mice (Figure 2C).

^1^H NMR spectroscopy is a reliable technique for the identification of metabolites in tissue extraction. The representative ^1^H NMR spectra assignments with chemical shifts for signals identified in the lipid extract of mice liver were shown in Figure 2D,E. The contents of hepatic lipid metabolites in db/db group were significantly higher than the control group (Table 1). Hepatic triglyceride levels declined significantly in db/db mice fed with leucine compared with the db/db group (Table 1). No significant changes in total cholesterol, free cholesterol or esterified cholesterol were observed. 

### 2.4. Leucine Supplementation Stimulated Hepatic AMPK and Inhibited Fatty Acid Synthase (FAS) Expression 

**Figure 3:**
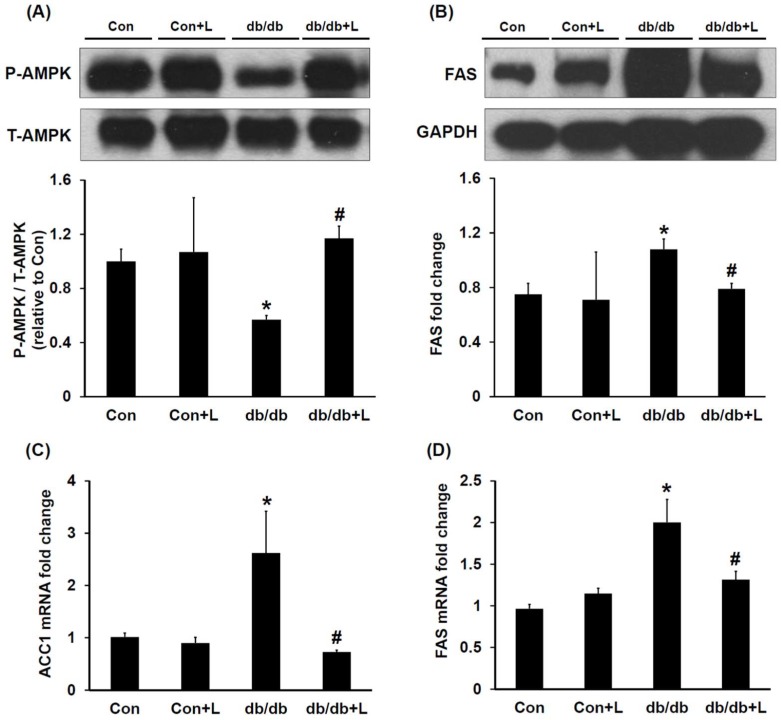
In db/db + Leu mice, AMPK was activated and the expression of hepatic fatty acid synthase (FAS) was reduced compared with that of db/db mice. Mice were sacrificed, and livers were harvested after 12 weeks of chew diet and normal drinking water. In mice treated with leucine, 1.5% *w*/*v* of leucine was added in the drinking water. (**A**) Hepatic phosphor-AMPK and AMPK levels were analyzed by Western blotting. The relative abundance of phosphorylated AMPK was calculated by Image J. (**B**) Relative abundance of the hepatic FAS was analyzed by Western blotting. The relative intensity of FAS was assessed by Image J. (**C**,**D**) qPCR analysis of the mRNA expression of the hepatic ACC1 and FAS. Data were expressed in fold relative to the expression in control mice. Data were presented as mean ± SEM, *n* = 5 in Con and Con + Leu (Con + L) groups; *n* = 6 in db/db and db/db + Leu (db/db + L) groups. * *p* < 0.05, Con vs. db/db mice; # *p* < 0.05, db/db vs. db/db + L mice.

To investigate if leucine supplementation stimulates hepatic AMPK activity and leads to reduced hepatic lipogenesis, we assessed the hepatic AMPK and lipogenesis key enzyme FAS by Western blotting. We found that the relative abundance of hepatic AMPK in db/db mice was about 50% lower than in db/db + Leu mice (Figure 3A). Interestingly, in association with AMPK activation, the expression of hepatic FAS in leucine-treated db/db mice was significantly suppressed (Figure 3B). In addition, the changes in Acetyl-CoA carboxylase 1 (ACC1) and FAS mRNA expression were consistent with changes in the respective protein expression levels (Figure 3C,D).

### 2.5. Leucine Supplementation Ameliorated Early DN 

**Figure 4:**
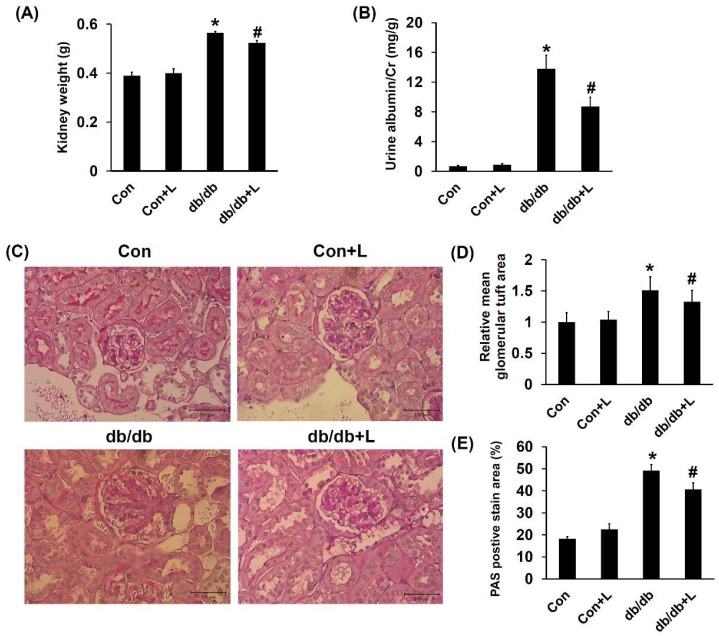

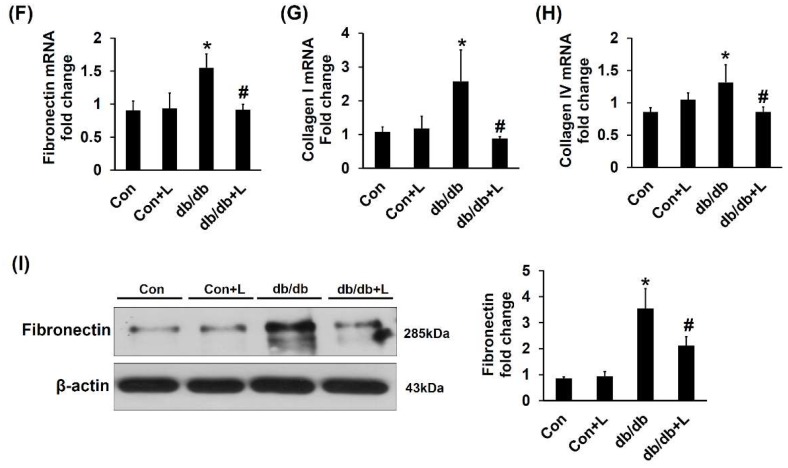
Diabetic glomerulosclerosis and albuminuria in db/db mice were attenuated by dietary leucine supplementation. Mice were sacrificed, and kidneys were harvested after 12 weeks of chew diet and normal drinking water. In mice treated with leucine, 1.5% *w*/*v* of leucine was added in the drinking water. (**A**) Absolute kidney weights were compared in various treatment groups. (**B**) Urine albumin-to-creatinine ratio of mice in various groups. (**C**) Photomicrographs illustrating PAS staining of glomeruli from mice in various treatment groups. (The scale bar shows 100μm) (**D**) The mean glomerular tuft area from 20 random glomeruli was analyzed, and data was presented with relative ratio to control mice. (**E**) The PAS staining-positive area in the glomeruli (dark red) relative to the total glomeruli area from 20 random glomeruli was analyzed. (**F**–**H**) qPCR analysis of the mRNA expression of genes encoding fibronectin (**F**), collagen I (**G**) and collagen IV (**H**). Data are expressed as fold relative to that of the control mice. (**I**) Renal cortex tissue lysates were also subjected to immunoblot analysis using specific antibodies against fibronectin. Protein expression levels were quantified by densitometry and normalized to β-actin levels. Data were presented as mean ± SEM, *n* = 5 in Con and Con + Leu (Con + L) groups; *n* = 6 in db/db and db/db + Leu (db/db + L) groups. * *p* < 0.05, Con vs. db/db mice; # *p* < 0.05, db/db vs. db/db + L mice.

Absolute kidney weight measurement showed that db/db mice exhibited higher kidney weight, and leucine partially reduced the weight changes (Figure 4A). Increased urine albumin to creatinine ratio is an early marker of DN; we found that the ratio was significantly elevated in db/db mice compared with Con mice and was significantly reduced in db/db + Leu mice (Figure 4B). Glomerular tuft area was used to assess glomerular hypertrophy. The glomerular tuft area was relatively larger in db/db mice but decreased in db/db + Leu mice (Figure 4D). Periodic acid-Schiff (PAS) staining clearly showed mesangial expansion and increased matrix protein accumulation in the glomeruli of db/db mice compared with that of Con and Con + Leu mice. The PAS staining area in glomeruli of db/db + Leu mice was significantly decreased compared with that of db/db mice (Figure 4C,E).

Expressions of matrix proteins in the renal cortex were further analyzed. RT-PCR analysis revealed that the expression of genes encoding fibronectin, type I collagen and type IV collagen were all significantly increased in db/db mice compared with that of Con mice. Interestingly, in db/db + Leu mice, the increased matrix gene expressions were attenuated (Figure 4F–H). In agreement with the mRNA expression results, Western blotting analysis showed that leucine supplement resulted in reduced fibronectin protein levels compared with that of db/db mice (Figure 4I). Taken together, the results indicated that dietary leucine supplementation significantly ameliorated early DN in db/db mice. 

### 2.6. Urinary Metabolite Profiles by LC-TOFMS-Based Metabolomics Analysis

**Figure 5:**
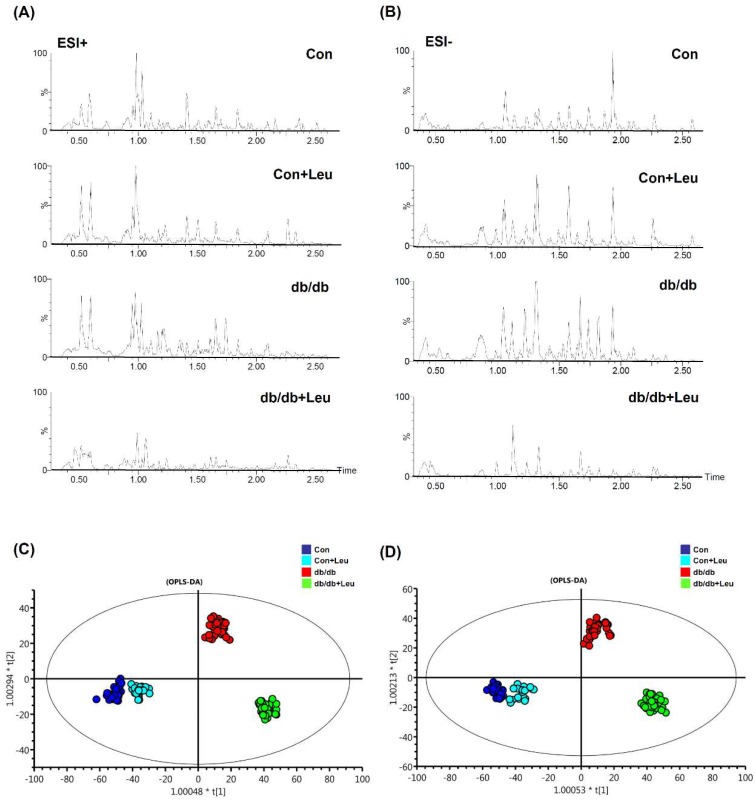
Liquid chromatograms of urine samples and orthogonal partial least squares discriminant analysis (OPLS-DA) of urine metabolomes. Mice were sacrificed, and urine samples were harvested after 12 weeks of chew diet and normal drinking water. In mice treated with leucine, 1.5% *w*/*v* of leucine was added in the drinking water. Urine samples were collected for LC-TOF-MS analysis, and features were acquired in electrospray positive-ion (ESI+) (**A**) and electrospray negative-ion (ESI−) (**B**) modes. OPLS-DA analysis generated by these features are shown for ESI+ (**C**) and ESI− (**D**) modes.

A global manner metabolites analysis using LC-MS, coupled with univariate and multivariate statistical analysis, was performed to generate the urine metabolomes among Con, Con + Leu, db/db, db/db + Leu groups. The representative base peak intensity chromatograms were obtained by scanning in electrospray positive-ion (ESI+) and electrospray negative-ion (ESI−) modes (Figure 5A,B). Orthogonal Partial Least Squares Discriminant Analysis (OPLS-DA) was further performed. The OPLS-DA score plots of ESI+ and ESI- modes (Figure 5C,D) showed that the clusters of Con and Con + Leu mice were quite closed, but the clusters between db/db and db/db + Leu mice were well-discriminated from each other.

**Figure 6:**
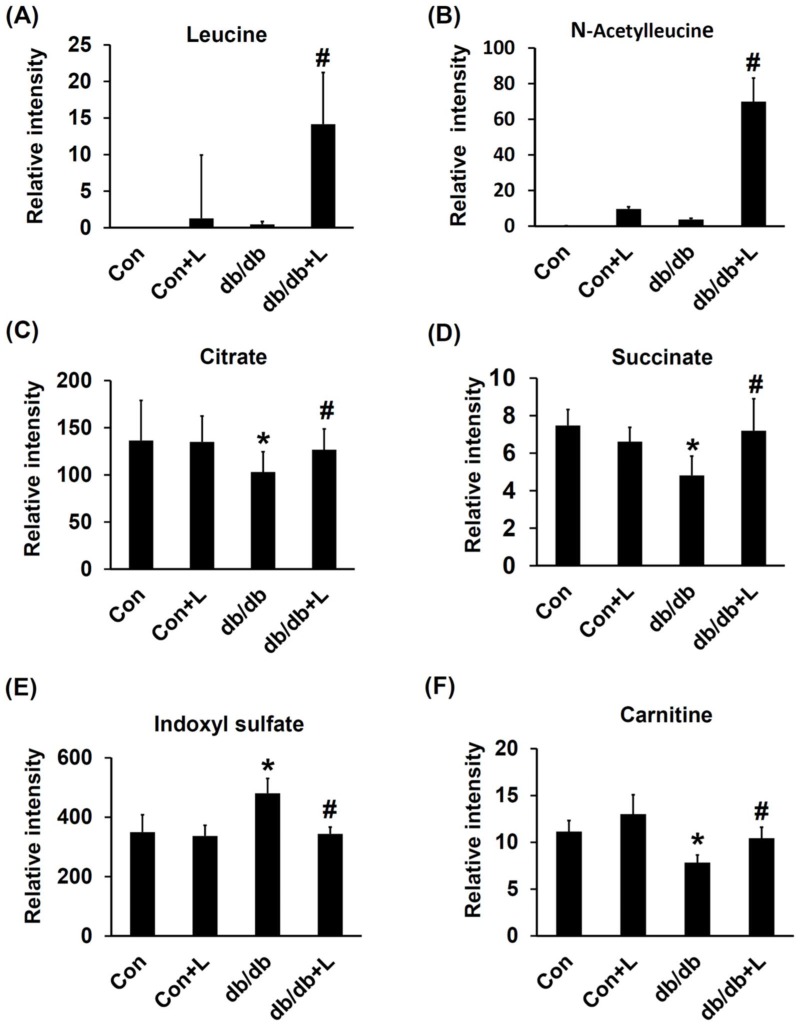
Urinary metabolites confirmed by MS/MS analysis in mice. Mice were sacrificed, and urine samples were harvested after 12 weeks of chew diet and normal drinking water. In mice treated with leucine, 1.5% *w*/*v* of leucine was added in the drinking water. The relative intensity of urinary leucine (**A**), acetyl-leucine (**B**), citrate (**C**), succinate (**D**), indoxyl-sulfate (**E**), and carnitine (**F**) of various groups are as shown. Data were presented as mean ± SEM. * *p* < 0.05, Con vs. db/db mice; # *p* < 0.05, db/db vs. db/db + L mice.

Metabolites that exhibited significant level variations (VIP score >2) in the db/db and db/db + Leu groups were searched against the Human Metabolome Database (HMDB) and KEGG databases. Six metabolites were selected and identified based on their chromatographic retention times and MS- and MS/MS information from the standards. The relative signal intensities of these targeted metabolites in the four groups are shown in Figure 6. The urinary levels of leucine and acetyl-leucine (down-stream metabolite) were significantly elevated in leucine-treated db/db mice (Figure 6A,B). The urine levels of citrate and succinic acid in db/db mice were reduced but restored to normal in db/db + Leu mice (Figure 6C,D). In addition, the urine levels of tryptophan downstream metabolites, indoxyl sulfate, was significantly lower in db/db + Leu mice than in db/db mice (Figure 6E). Moreover, the urine levels of carnitine were decreased in db/db mice compared with that of Con mice. In db/db + Leu mice, the urine carnitine levels were reverted toward normal (Figure 6F).

### 2.7. Leucine Supplement Activated p-AMPK Expression and Autophagy in the Kidney of db/db Mice

**Figure 7:**
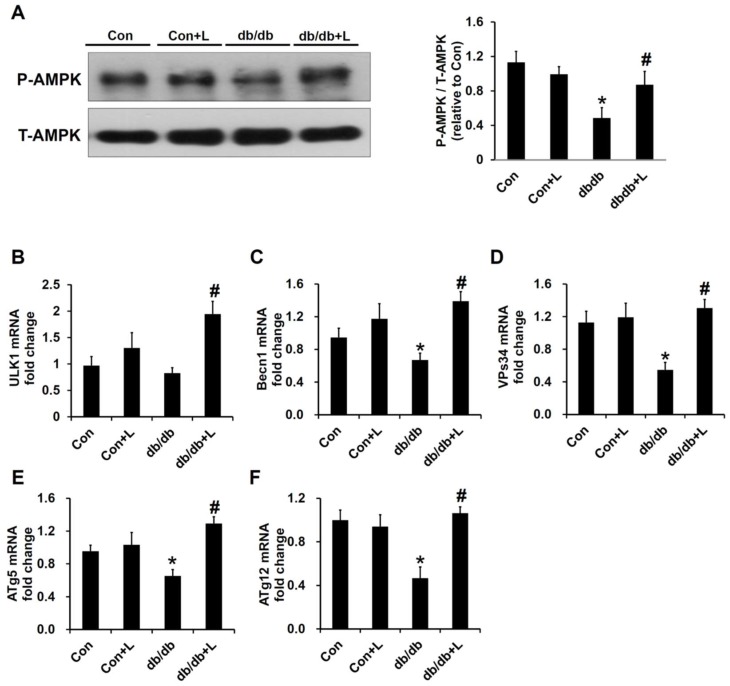
In db/db + Leu mice, phospho-AMPK and autophagy genes expression were increased compared with that of db/db mice. Mice were sacrificed, and kidneys were harvested after 12 weeks of chew diet and normal drinking water. In mice treated with leucine, 1.5% *w*/*v* of leucine was added in the drinking water. (**A**) Renal cortex tissue lysates were subjected to immunoblot analysis using specific antibodies against phospho-AMPK and total-AMPK. The relative abundance of phosphorylated AMPK was calculated by Image J. (**B**–**F**) qPCR analysis of the mRNA expression of the autophagy related genes, including ULK1 (**B**), Becn1 (**C**), VPs34 (**D**), ATg5 (**E**), and ATg12 (**F**). Data were expressed in fold relative to the expression in control mice. Data were presented as mean ± SEM, *n* = 5 in Con and Con + Leu (Con + L) groups; *n* = 6 in db/db and db/db + Leu (db/db + L) groups. * *p* < 0.05, Con vs. db/db mice; # *p* < 0.05, db/db vs. db/db + L mice.

AMPK activation has been thought to be one important target of leucine treatment in recent studies [14,16]. In the renal cortex extract, the protein expression levels of p-AMPK were decreased in db/db mice, and the expression was significantly increased in db/db + Leu mice (Figure 7A). Phosphorylation of Unc-51-like autophagy activating kinase 1 (ULK1) by AMPK is a necessary step to induce autophagy, and impaired autophagy has been shown to promote the development of DN [17]. RT-PCR analysis showed that the mRNA expressions of ULK1, Becn1, VPs34, Autophagy related 5 (ATg5), and ATg12 were all significantly reduced in db/db mice compared with control mice, and the levels were reversed toward the control levels in db/db + Leu group (Figure 7B–F).

## 3. Discussion

Diet rich in carbohydrates and/or fat is associated with metabolic disorders. Single leucine supplementation has been shown to ameliorate insulin resistance and hepatic lipogenesis in HFD-induced obese mice [14]. In this study, we further demonstrated that in db/db mice (most commonly used animal model of type II DM), dietary leucine supplementation can also attenuate insulin resistance, hepatic steatosis and early DN. AMPK activation by leucine may be the important mechanism underlying these beneficial effects. 

Consistent with a previous report in HFD mice by Macotela et al. [14], we found that daily leucine supplementation (1.5% *w*/*v* leucine) leads to approximate 1.5-fold increase in plasma leucine concentrations. The effects of leucine supplementation on the body weight of HFD mouse model studies were somewhat controversial [14,18,19]. In our results, leucine supplementation had no obvious influence on the body weight neither in control mice nor in db/db mice over a 12-week-period. Although BW was not reduced by leucine treatment in our results, HOMA-IR, a hallmark of insulin resistance, was markedly improved in leucine-supplemented db/db mice. The effect of leucine supplementation on insulin resistance has been shown to be due to increased energy expenditure, reduced inflammation of the adipose tissue, and decreased adiposity in HFD obese mice [14,19,20]. In addition, despite the mTOR activation, leucine supplementation of HFD mice improved glucose tolerance, and the effect was correlated with the enhancement of phosphorylation/activation of the insulin receptor, Insulin receptor substrate 1 (IRS-1) and AKT in major metabolic organs, such as muscle, fat, and liver [14]. Moreover, leucine supplementation was also shown to improve insulin-induced Protein kinase B (PKB) phosphorylation in adipose tissue explants obtained from db/db mice, suggesting that leucine may have therapeutic benefit to improve disease-associated insulin-resistance in adipocytes [21].

In db/db mice, with 12 weeks of leucine supplementation, the hepatic steatosis and liver weight was significantly reduced compared with that of db/db mice and was ascribed mainly to reduced hepatic lipid accumulation. In this study, ^1^H NMR-based hepatic lipidomic analysis was performed to analyze the hepatic lipid composition. As was shown by ^1^H NMR spectra, hepatic lipids, including total cholesterol, free cholesterol, esterified cholesterol, and TG were all elevated in db/db mice compared with that of the control mice. Hepatic lipids were reduced in db/db + Leu mice, with TG reduction being the most significant. The results were consistent with a significant reduction in plasma TG level in db/db + Leu animals. The mechanisms underlying the amelioration of hepatic steatosis by leucine supplementation are thought to be related to reduction of lipogenic genes expression, AMPK activation and liver inflammation [14,16]. In accordance with these trends, we also found that leucine supplementation stimulated hepatic AMPK activity and reduced the expression of hepatic lipogenic target enzyme FAS in db/db + Leu mice. Leucine has been shown to modify SIRT1-AMPK activation, which ameliorates mitochondrial dysfunction and regulates lipogenesis and glucose tolerance in HFD mice [10,22,23]. On the other hand, a 7-day leucine deprivation was shown to activate AMPK and improve metabolic health in obese mice [24]. Further study using HFD AMPK knockout mice may help to clarify the controversy.

Although isolated leucine supplementation has been shown to improve body mass composition (fat to muscle ratio), glucose tolerance, and hepatic steatosis in many studies, on the contrary, many other studies also showed that BCAA mixture aggravated metabolic disorders in HFD mice, especially through mTORC1 pathway activation [6]. Recent studies by Cummings et al. further showed that BCAA restriction in diet can improve metabolic health in both lean and obese mice [8,9]. The reason for these conflict effects may be related to the different metabolic effects between single leucine and BCAA mixture supplementation. Especially in the study by Cummings et al., mice fed with isolated low-leucine diet surprisingly developed more white adipose tissue mass in skin and epididymal fat pads [8]. Although the molecular mechanism was not elucidated in the study, the phenomenon reflected that leucine alone had metabolic benefits but not BCAA mixture. The discrepancy results between single leucine and BCAA mixture supplements in obese mice warrant further study. 

In addition to the metabolic benefits of leucine in db/db mice, we also examined if early DN in db/db mice can be ameliorated by leucine supplementation. We found that diabetic renal hypertrophic change, glomerulosclerosis and albuminuria were improved by leucine supplementation. To our knowledge, this study is the first to show that dietary leucine supplementation can ameliorate DN in db/db mice. We suggested that AMPK activation and energy metabolism improvement in the kidneys of db/db mice may be involved. 

Diabetic renal hypertrophy was associated with reduction of AMPK phosphorylation and could be reversed by metformin, 5-Aminoimidazole-4-carboxamide ribonucleotide (AICAR), or other AMPK activators [25,26]. In addition, diabetic glomerulosclerosis is characterized by diffuse mesangial matrix expansion and is largely dependent on the TGF-β/Smad signaling pathway. AMPK activation has been shown to inhibit the upregulation of Smad4 and reduce glomerulosclerosis [27]. In this regard, our finding that dietary leucine supplementation activated AMPK in the renal cortex of the db/db mice is of pathophysiological significance. Autophagy is a catabolic process that degrades damaged proteins and organelles in mammalian cells and plays a critical role in maintaining cellular homeostasis [28]. The accumulation of proteins and organelles damaged by hyperglycemia and other diabetes-related metabolic changes is highly associated with the development of DN [17,29]. Activation of AMPK has been shown to promote autophagy [30]; therefore, the effects of leucine on autophagy in the kidney were further investigated. We found that the mRNA levels of autophagy-related molecules, such as ULK-1, Becn1, VPs34, ATg5, ATg12 were all reduced in the kidney of db/db mice compared with that of the normal control. In db/db + Leu mice, the mRNA levels of the aforementioned protein species were significantly increased to an extent comparable to that of the control mice.

As urine metabolomics is a tool to realize the metabolic changes and signature of DN, urine metabolomics analysis was used to explore the effects of leucine supplementation. Earlier urine metabolomics analysis showed progressively reduced urinary levels of citrate and succinate with age in db/db mice suggesting a perturbation of the TCA cycle and the development of DN [31,32]. We found that the reduced urinary levels of citrate and succinate in db/db mice were significantly reversed by leucine supplementation, implying that the disturbance of the TCA cycle in DN is probably attenuated. In addition, disturbance of indoxyl sulfate, an important down-stream metabolic in the catabolic pathway of tryptophan metabolism, has been thought to be associated with deteriorated renal function [33]. Our results showed that the increased level of urinary indoxyl sulfate in db/db mice was reduced in db/db + Leu mice. Metabolic disturbance of carnitine is a common phenomenon associated with DM due to disturbed fatty acid oxidation, and reduced urinary carnitine in the diabetic animals has been reported in our prior studies [34]. We further showed that in db/db + Leu mice, the urinary levels of carnitine were increased. All the above findings suggested that the disturbance of the TCA cycle in db/db mice was improved by leucine supplementation, and we suggested that AMPK activation by leucine is, or at least in part, responsible.

In conclusion, dietary leucine exhibits metabolic benefits in obese, diabetic db/db mice, including the attenuation of insulin resistance, hypertriglyceridemia, hepatic lipid accumulation, disturbance of the TCA cycle metabolism and urinary carnitine levels. Moreover, the autophagy in the renal cortex was increased, and DN was improved in leucine-treated db/db mice. All the above benefits seen in db/db + Leu mice may be partially explained by the AMPK activation. Although BCAAs associated with mTORC1 activation have detrimental metabolic effects in obese mice, many studies also showed that single leucine supplementation has potential metabolic benefits. From current evidence, AMPK activation may be an important key issue. To clarify the contrary, further study to investigate the effects of BCAA mixture and isolated leucine on the mTORC1, SIRT1-AMPK interaction in vivo and in vitro is needed.

## 4. Materials and Methods

### 4.1. Ethics Statement

Animal experiments were carried out in accordance with the guidelines issued by the Institutional Animal Care and Use Committee, Chang Gung University. This study was approved by the Animal Care and Use Committee of Chang Gung University, and basic standards of laboratory animal care were followed (Approval number: CGU12-106; Project identification code: CMRPG3D0931).

### 4.2. Reagents and Chemicals

All aqueous solutions were prepared using deionized water (with a resistance of 18.2 MΩ·cm) from a Millipore MillQ system. Leucine and extra pure-grade ammonium formate (98–100%) were purchased from Sigma (St Louis, MO, USA). Fasting insulin was measured by enzyme immunoassay using 10-μL aliquots of plasma with a Mice Insulin ELISA kit (Mercodia, Uppsala, Sweden). Measurements of fasting plasma glucose, cholesterol and triglyceride levels were performed using an auto-analyzer (VITROSR 350 Chemistry System, Ortho-Clinical Diagnostics, Inc., Raritan, NJ, USA). Antibodies to AMPK, phosphor-AMPK were purchased from Cell Signaling Technology (Danvers, MA, USA) and antibody to fatty acid synthase (FAS), fibronectin, GAPDH and β-actin were purchased from Abcam (Cambridge, MA, USA).

### 4.3. Animal Model and Treatments

Male B6.BKS-Leprdb mice (db/db mice) ranging from 6–8 weeks old were obtained from Taiwa Instrument Co Ltd., Taipei, Taiwan. The db/db mice are typical models resembling morbid obesity and spontaneous T2DM. Male B6 BKS (*m+*/*m+*) mice were used as control mice. Mice were kept in an animal house at 22 ± 2 °C and 55 ± 10% relative humidity and maintained under a 12-h light-dark cycle. Water and normal rodent chow (Sani-Chips; Montville, NJ, USA) were provided ad libitum. Control mice or db/db mice were supplemented with or without leucine via drinking water. In mice treated with leucine, 1.5% *w*/*v* of leucine was added in the drinking water as dietary supplement [14].

Urine and plasma samples were collected from control (Con), control + leucine (Con + Leu), db/db, db/db + leucine (db/db + Leu) after 12 weeks of treatment. Mice were transferred to metabolic cages 24 h before urine collection. Urine was collected from each animal over a 24-h period. Food and water consumption during the same period were also recorded. Urine samples were filtered through a 0.2-μm filter and stored at −80 °C until they were analyzed. Blood samples (100 μL) were collected from punctured facial venous sinus from animals fasted overnight and stored at −80 °C.

Animal kidneys and liver tissue were quickly removed and snap frozen in liquid nitrogen and stored at −80 °C until further analysis. The middle third of the right kidney and right liver lobe was fixed in 4% paraformaldehyde and embedded in paraffin for morphology evaluation and immunohistochemistry.

### 4.4. Evaluation of Diabetic Glomerulosclerosis

Periodic acid-Schiff (PAS) staining was used for kidney sections to evaluate the degree of diabetic glomerulosclerosis. Twenty individual glomeruli in high-power fields (magnification, 200×) per kidney were analyzed. The mean glomerular tuft cross-sectional area was analyzed. The percentage area occupied by extracellular matrix in the mesangial area (dark red color) was analyzed using computer-assisted image analysis software (Meta-Morph, version 4.6, Universal Imaging Corporation, Downingtown, PA, USA).

### 4.5. Western Blotting Analysis

Total cellular protein from kidney and liver was extracted as previously described [35]. Equal amounts of proteins were mixed with an equal volume of reducing SDS sample buffer and boiled at 95 °C for 5 min. Protein samples (20 μg) were resolved by 10% SDS-PAGE and electroblotted on nitrocellulose membranes (Bio-Rad, Hercules, CA, USA). After electroblotting, nonspecific binding was blocked with 5% nonfat milk. The membrane was incubated with primary antibodies overnight at 4 °C and incubated with horseradish peroxidase–conjugated secondary antibodies for 1 h at room temperature. Primary antibodies against the following proteins were used at 1:1000 dilutions unless otherwise indicated: fibronectin, phosphor-AMPK, AMPK, FAS, and β-actin (1:20,000). Immunoreactive bands were visualized using enhanced chemiluminescence (Amersham, Arlington Heights, IL, USA) as previously described.

### 4.6. Quantitative Real-Time PCR Analysis

Real time-PCR was performed using total RNA isolated from the liver and kidney samples in an ABI-Prism 7000 with SYBR Green I as double-stranded DNA-specific dye, according to the manufacturer’s instructions (PE-Applied Biosystems, Cheshire, UK). Expression of GAPDH or 18S mRNA was used as an internal control. Primer sequences used are listed in Table S1. Primers were constructed to be compatible with a single RT-PCR thermal profile (95 °C for 10 min, and 40 cycles of 95 °C for 30 s, and 60 °C for 1 min). The fold change in gene expression was determined with 2^-ddct^ method for the Con, Con + Leu, db/db, db/db + Leu groups.

### 4.7. Liquid Chromatography/Time-of-Flight Mass Spectrometry (LC/TOF MS)–Based Metabolomics Analysis of Urine Samples 

Urine was normalized with water to contain a creatinine concentration of 50 ug/mL and was filtered with 0.2 μm filter (millipore) to remove particles. The clear diluent was analyzed using LC/TOFMS (Waters Corp.). The chromatographic separation was achieved on a BEH C18 column (100 mm × 2.1 mm, particle size of 1.7 um; Waters Corp., Milford, MA, USA) using an ACQUITY TM UPLC system. The gradient profile was as follows: linear gradient 1–48% B, 2 min; 98% B, 0.5 min; and keep 98% B 1 min. The column was then re-equilibrated for 1.8 min. QC samples were prepared from mixed urine sample and analyzed during the analytical runs after every 15th sample. The eluent was analyzed via high-definition, TOFMS (SYNAPT G1, Waters Corp., Milford, MA, USA ) in both positive (ESI+) and negative (ESI−) mode. The optimized parameters were as follows: voltage at 35 V; capillary voltage at 3 kV in positive mode, and 1 kV in negative mode; desolyation temperature at 300 °C; source temperature at 80 °C; and gas flow at 700 L/h. 

For structural identification, we used identical chromatographic conditions that were used in the profiling experiment for the metabolite standards.

### 4.8. Liver Sample Extraction for Lipidomic Analysis by ^1^H-NMR

Mice liver extracts were prepared using a modified Folch’s method. Liver tissue (200 mg) was homogenized in 3 mL ice cold Folch solution (methanol/chloroform, 2:1, *v*/*v*) for 30 s using IKA T10 basic ULTRA-TURRAX homogenizer (Heidolph Instruments, Schwabach, Germany) and diluted with 0.8 mL double-distilled water. The mixture was vortexed for 1 min, followed by centrifugation at 2000 *g* for 10 min at room temperature. The organic phase was removed and dried in a nitrogen evaporator to obtain the lipid extract. The lipid extract was dissolved in 600 μL chloroform-d containing 0.1% tetramethylsilane (TMS), an NMR chemical shift reference compound. TMS was employed for NMR chemical shift reference and concentration calibration.

The CDCl3 provided a field frequency lock, and TMS provided a chemical shift reference (δH 0.0). ^1^H NMR spectra were acquired on a Bruker Avance III HD 600 MHz spectrometer, operating at temperature of 300 K using a Bruker TCI cryo-probehead probe (Bruker Biospin, Rheinstetten, Germany). Samples were transferred to NMR sample jet system, and NMR spectra were acquired using the one-dimensional pulse sequence (Zg30). For each sample, 64 transients were collected into 64 K data points using a spectral width of 12,000 Hz with a relaxation delay of 4 s. A line-broadening function of 0.3 Hz was applied to all spectra prior to Fourier transformation (FT). 1H NMR spectra were manually corrected for phase and baseline distortions using TOPSPIN (version 3.2, Bruker Biospin) and referenced to the TMS signal (δH 0.0).

### 4.9. Statistical Analysis

All the data were expressed as mean ± SE. One-way analysis of variance (ANOVA) was conducted for multiple-group comparisons, and an LSD or Tukey’s post hoc-test was used to evaluate the significance of differences between group means. In all analyses, *p* ≤ 0.05 were considered significant.

## Figures and Tables

**Table 1:** Concentration of lipid metabolites in the liver.

Lipid	Control (*n* = 5)	Con + Leu (*n* = 5)	db/db (*n* = 6)	db/db + Leu (*n* = 6)
Total cholesterol (μmol/g)	1.86 ± 0.22	2.07 ± 0.11	3.12 ± 0.29 *	2.46 ± 0.11
Free cholesterol (μmol/g)	0.48 ± 0.09	0.68 ± 0.08	1.29 ± 0.21 *	0.86 ± 0.07
Esterified cholesterol (μmol/g)	1.17 ± 0.30	1.22 ± 0.18	0.98 ± 0.05	0.98 ± 0.11
TG (μmol/g)	5.65 ± 0.42	14.81 ± 2.63	37.38 ± 3.30 *	24.4 ± 1.53 ^#^

Concentrations of lipid metabolites in the mice liver were calculated. Data were presented as mean ± SEM. * *p* < 0.05, Con vs. db/db mice; # *p* < 0.05, db/db vs. db/db + Leu mice.

## Supplementary Materials

The following are available online at http://www.mdpi.com/1422-0067/19/7/1921/s1, Table S1: Forward and reverse primers used for quantitative real-time reverse transcriptase PCR.
