# Supplementary material for: Dietary Leucine Supplement Ameliorates Hepatic Steatosis and Diabetic Nephropathy in db/db Mice
Source: Int J Mol Sci. 2018 Jun 30;19(7):1921. doi: 10.3390/ijms19071921 (PMC6073714; doi:10.3390/ijms19071921)
Supplement: Supplementary file 1 [file ijms-19-01921-s001.pdf]

Supplement 1

Table S1. Forward and reverse primers used for quantitative real-time reverse transcriptase PCR

Gene	Type	Sequences (5′ → 3′)
ACC1	Forward	TGACAGACTGATCGCAGAGAAAG
	Reverse	TGGAGAGCCCCACACACA
FAS	Forward	GCTGCGGAAACTTCAGGAAAT
	Reverse	AGAGACGTGTCACTCCTGGACTT
Fibronectin	Forward	TGTGACCAGCAACACGGTG
	Reverse	ACAACAGGAGAGTAGGGCGC
Collagen I	Forward	AAGGGGTCTTCCTGGTGAAT
	Reverse	GGGGTACCACGTTCTCCTC
Collagen IV	Forward	CTCTGGGGACAACATCCG
	Reverse	TCTTCTCATGCACACTTGGC
ULK1		Mm00437238_m1
Becn1		Mm01265461_m1
VPs34		Mm00619489_m1
ATg5		Mm01187303_m1
ATg12		Mm00503201_m1
GAPDH		Mm99999915_g1
18S rRNA		Mm03928990_g1
